# The silent sinus syndrome: protean manifestations of a rare upper respiratory disorder revisited

**DOI:** 10.1186/1476-7961-11-5

**Published:** 2013-12-09

**Authors:** Denisse E Guillen, Paulette M Pinargote, Juan C Guarderas

**Affiliations:** 1 Department of Otolaryngology, Mayo Clinic, 4500 San Pablo Road, Jacksonville, FL 32224, USA; 2 Department of Otorhinolaryngology, Mayo Clinic, 4500 San Pablo Road, Jacksonville, FL 32224, USA

## Abstract

Silent Sinus Syndrome (SSS) is known to be a rare clinical condition, characterized by spontaneous and progressive enophthalmos and hypoglobus associated with atelectasis of the maxillary sinus and alteration of the orbital floor. Most of the patients with this syndrome present with ophthalmological complaints without any nasal sinus symptoms, and it typically has a painless course and slow development, ergo the term “silent.” Here we present a case report of a patient with occasional coughing spells as the presenting symptom of Silent Sinus Syndrome, which has not been previously described in the literature. The CT scan findings suggested chronic rhinosinusitis. The radiological findings were suggestive of maxillary sinus hypoplasia, with evidence of maxillary sinus atelectasis. Awareness of this syndrome is important for specialists who work with nasal sinus disease, since its management is different than chronic rhinosinusits.

## Background

Silent Sinus Syndrome (SSS) is known as a rare clinical condition, characterized by spontaneous and progressive enophthalmos and hypoglobus [1] associated with atelectasis of the maxillary sinus and alteration of the orbital floor. This syndrome was first described in 1964 by Montgomery [2], but it was named Silent Sinus Syndrome in 1994 by Soparkar et al. [3]. Most of the patients with this syndrome present with ophthalmological complaints without any nasal sinus symptoms, and it typically has a painless course [3] and slow development, ergo the term “silent.” We review the concepts of SSS and review the two most likely mechanisms of this condition.

It’s very common that these patients first present to ophthalmology [4] due to the syndrome’s typical constellation of progressive enophthalmos and hypoglobus. SSS typically presents unilaterally [5], with a slight predominance for presenting on the right maxillary sinus (57%) [6], and its development is gradual and progressive. The physical exam shows some degree of orbital asymmetry, with deepening of the superior orbital sulcus and the consequent hypoglobus. Some other ophthalmological signs can be eyelid retraction, lid lag, and lagophthalmos [7]. Occasionally, exophthalmos of the contralateral uninvolved eye is reported [8]. Although the visual function is typically unaffected, a few patients have reported alterations in ocular motility or muscle imbalance producing diplopia [9].

### Radiographic findings

The computed tomography (CT) scans of the nose and paranasal sinuses typically show opacification of the maxillary sinus and inferior bowing of the orbital floor [10]. The sinus can be developed or hypoplastic but is opacified, and the infundibulum is obstructed. This obstruction is usually caused by a lateral retraction of the uncinate process with its apposition in the inferiomedial part of the orbit [11]. Sanchez et al. described an image of a “pseudo-pneumo-orbit” that can also be seen due to air trapped under the upper eyelid [12]. Both CT and magnetic resonance imaging (MRI) scans allow physicians to perform a SSS diagnosis, but CT scans are considered the gold standard diagnostic method because they provide a better view of the anatomical changes of SSS that are needed for its diagnosis and for differentiation from other conditions [13].

### Management and treatment

Treatment should address the obstruction of the sinus and the resultant ocular consequences. Treatment consists of reaeration of the atelectatic sinus by endoscopic sinus surgery. All authors agree that sinus pathology should be treated endoscopically as the first step of the treatment [14].

Limited antrostomy typically results in a release of negative sinus pressure and re-expansion of the collapsed cavity leading to reduction of enopthalmus [15]. A wide antrostomy prevents future reobstructions, and good reaeration of the sinus helps to avoid recurrent enophthalmos [16].

Timing for management of the orbital floor is still under debate. As suggested by some authors, orbital floor reconstruction must be performed simultaneously with sinus treatment [17]. Other authors think that only drainage of the sinus should be enough [18]. According to Cardesin et al., the need for orbital floor repair depends on the severity of the diplopia, the degree of the cosmetic alterations, and the postsurgical evaluation of the sinus [19].

## Case presentation

**Figure 1:**
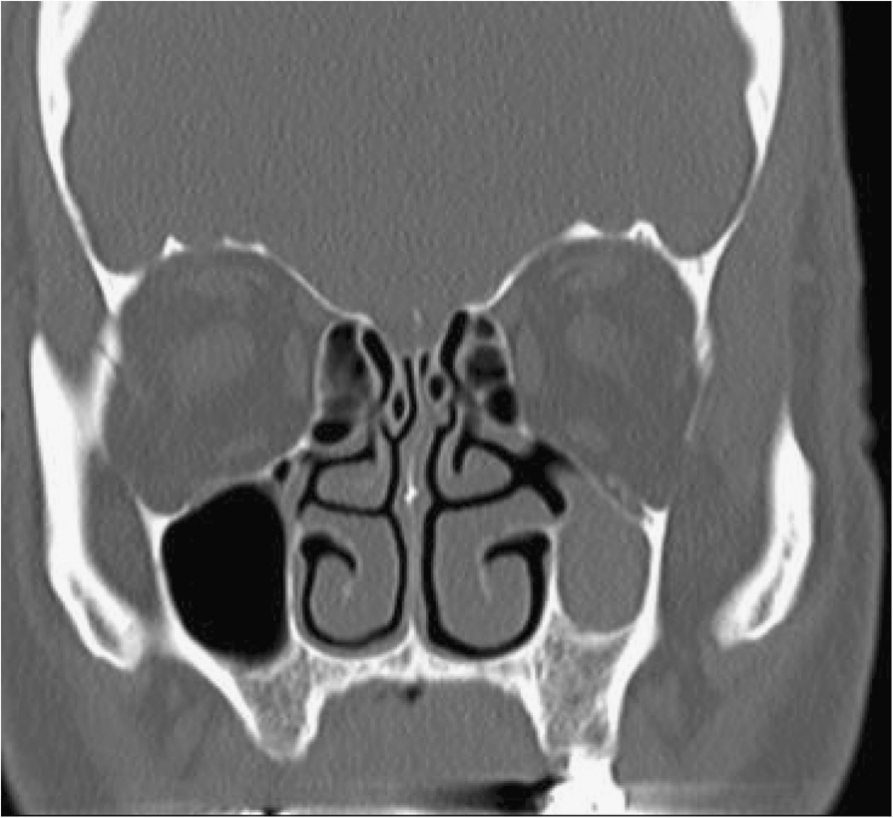
Examination reveals an asymmetrically smaller and completely opacified left maxillary sinus with left-sided periosteal thickening, as well as lateral bowing/bone remodeling of the uncinate process.

**Figure 2:**
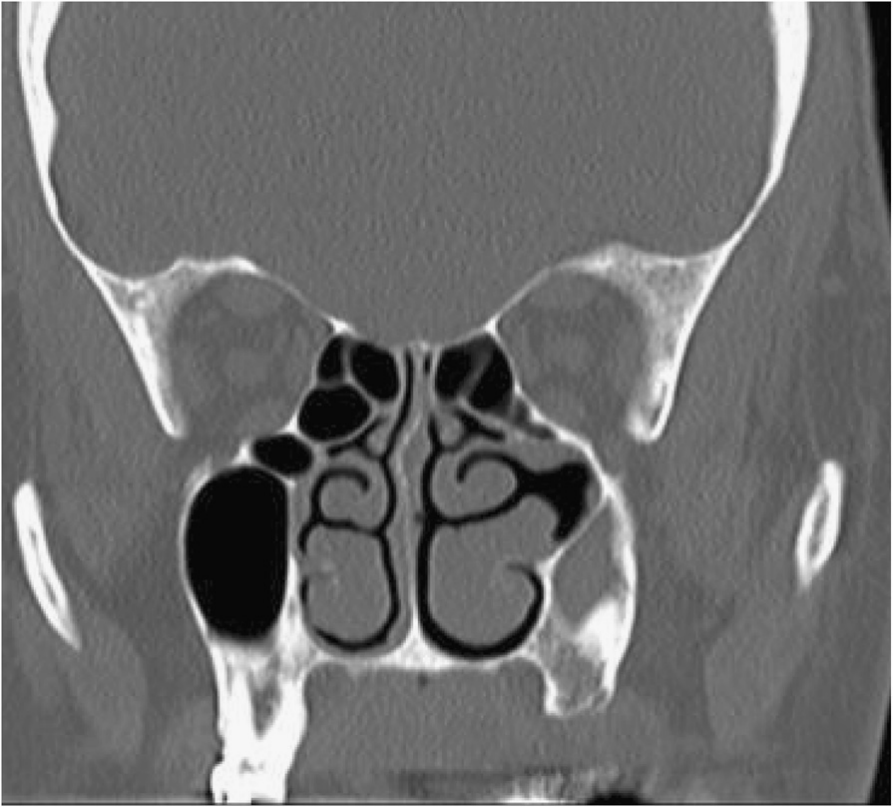
**The left maxillary sinus is smaller and opacified.** The middle meatus space is wide and the medial wall of the sinus is lateralized with contact with the lateral wall of the maxillary sinus.

A 66-year-old gentleman, non-smoker, with no known allergies or significant respiratory medical history, presented to the Allergy Medicine service with a chief complaint of cough. He describes that he suffered a respiratory infection approximately three months prior to the visit. After it was treated, the majority of symptoms resolved, but the cough and coughing spells persisted with post-nasal drainage and clearing of the throat. The CT scan revealed an asymmetrically smaller and completely opacified left maxillary sinus with left-sided periosteal thickening as well as lateral bowing/bone remodeling of the uncinate process. The opacified left maxillary sinus had hyperdensities which could represent chronic dense secretions (Figure 1). The left ostiomeatal complex was occluded (Figure 2). The initial diagnosis was chronic sinusitis. He was prescribed antibiotics for 10 days, was advised to have a new sinus CT scan, and was referred to otorhinolaryngology.

Two months later when the patient presented to otorhinolaryngology for follow-up, there had been no clinical changes. The physical exam showed some asymmetry of both eyes with mild hypoglobus of the left eye. His nose had a moderate anterior septal deformity to the left with mucoid drainage. He was unaware of vision changes, but testing demonstrated double vision when looking to the extreme right. The rest of the exam was normal.

The new sinus CT scan showed no significant change. The interpretation was a persistent opacified hypoplastic left maxillary sinus with obstructed left ostiomeatal unit with lateralization of the uncinate process. The pattern was consistent with type 2 maxillary sinus hypoplasia. A sinonasal endoscopy confirmed the previous imaging findings.

The patient underwent a turbinoplasty and endoscopic sinus surgery (antrostomy) to address the total opacification of his left maxillary sinus. The nasal sinus symptoms and cough resolved after the procedure. Correction of the enopthalmos was not necessary.

## Discussion

SSS has two main theorized mechanisms: maxillary sinus atelectasis (MSA)—which could be idiopathic, post-traumatic, or post-surgery—or maxillary sinus hypoplasia (MSH).

Chronic rhinosinusitis (CRS) has a prevalence of 13.4% in adults older than 18 years of age, according to a national health survey conducted in 2008 [20]. The causes and classification of CRS have recently been reviewed by Hamilos [21]. Causes of CRS are frequently anatomical and include septal deformity, Haller’s cells, paradoxical middle turbinate, and agger nasi cell. A hypoplastic maxillary sinus, an atelectatic maxillary sinus, and silent sinus syndrome are infrequently recognized causes of CRS. A description of these entities is reviewed in this paper.

Maxillary sinus hypoplasia (MSH) is an infrequent congenital anomaly that Bolger et al. [22] noted in 10.4% of 202 consecutive CT scans reviewed. MSH has been classified as type 1 when there is a normal uncinate process and a defined infundibular passage. Type 2 has a hypoplastic or absent uncinate process with an opacified affected sinus, and type 3 has an absent uncinate process and profound hypoplasia of the sinus. This classification has been supported by Erden (Table 1) [23].

**Table 1:** **Types of maxillary sinus hypoplasia **[23]

**Maxillary sinus hypoplasia type**	**Characteristics**
Type 1	Normal Uncinate. Defined infundibular passage
Type 2	Hypoplastic or absent uncinate. Opacified sinus
Type 3	Absent Uncinate. Profound hypoplasia of the sinus

Chronic maxillary atelectasis (CMA) is a term that describes a persistent decrease in the sinus volume from inwardly bowing antral walls [24]. In a 1997 study that spanned over ten years at the Massachusetts Eye and Ear Infirmary, 22 individuals were diagnosed with CMA, and their literature review found 25 additional individuals who met their criteria. Their criteria included: sinus opacification on CT scans or X-rays lasting more than 3 months and/or tenacious mucus secretions filling the antrum in addition to lateral displacement of the medial infundibular wall (MIW).

CMA, in reference to the Massachusetts Eye and Ear work, was differentiated in 3 stages based on the anatomical changes: Stage 1 (membranous deformity) where there is a lateralization of the maxillary fontanel, Stage 2 (bone deformity) where there is inward bowing of one or more osseous walls of the maxillary antrum, and Stage 3 (clinical deformity) where enophthalmus, hypoglobus, and/or midfacial deformity is noted (Table 2) [24].

**Table 2:** **Stages of chronic maxillary atectasis **[24]

	**Characteristics**
Stage 1	Membranous deformity where there is lateralization of the maxillary fontanel
Stage 2	Bone deformity where there is inward bowing of one or more osseous walls of the maxillary antrum
Stage 3	Clinical deformity with enophthalmus, hypoglobus, and/or midfacial deformity is noted.

In this series of 22 patients, 19 had some degree of sinus symptoms and five had findings of hypoglobus.

Silent sinus syndrome is a very uncommon clinical entity [25]. The pathophysiology of this syndrome remains unanswered in part because there is rarely a pre-symptomatic CT scan that can be used to review the stages of the process. A hypothesis for the pathophysiology is that hypoventilation of the sinus due to obstruction of the osteomeatal unit [26] creates a negative pressure [27] that leads to atelectasis [28] of the sinus with a downward displacement of the orbital floor [29]. There is disagreement over whether the obstruction of the osteomeatal unit (OMU) is caused by hypoplasia and/or if there are any cases where a normally developed sinus due to trauma, surgery, or other cause can be obstructed and consequently develop atelectasis and SSS.

## Conclusion

In this paper, we have reviewed the clinical and radiological presentation of SSS. Patients with SSS most often present to ophthalmology practices due to complaints of facial or ocular asymmetry such as hypoglobus or enophthalmos with little or no nasal sinus symptoms [30]. However, these patients may occasionally also present to otorhinolaryngology or allergy medicine, with nasal sinus symptoms suggestive of sinusitis. The mechanism of the development of SSS has been thought to be atelectasis of the maxillary sinus with or without the presence of maxillary sinus hypoplasia, especially in type 2.

Our patient presented to otolaryngology and allergy medicine for evaluation of a chief complaint of chronic cough with occasional coughing spells which have not been described in the literature as a form of presentation of Silent Sinus Syndrome. The CT scan findings suggested CRS. The radiological findings were suggestive of MSH, and there was evidence of MSA.

The differentiation of SSS from CRS is important since sinus surgery is the procedure of choice and clear knowledge of this anatomy is very important for the surgeon to avoid entering into the orbit and since medical management alone is unlikely to produce a positive result. In surgery, the use of an image-guidance system can also help to avoid complications.

## Consent

Written informed consent was obtained from the patient for publication of this Case Report and any accompanying images. A copy of the written consent is available for review by the Editor-in-Chief of this journal.

## Abbreviations

CMA: Chronic maxillary atelectasis; CRS: Chronic rhinosinusitis; CT: Computed tomography; MIW: Medial infundibular wall; MRI: Magnetic resonance imaging; MSA: Maxillary sinus atelectasis; MSH: Maxillary sinus hypoplasia; OMU: Ostiomeatal unit; SSS: Silent sinus syndrome.

## Competing interests

JG serves on the editorial board for *Clinical and Molecular Allergy.* The authors declare no other conflicts of interest.

## Authors’ contributions

DG: conception and design; acquisition of data; analysis and interpretation of data; drafting the manuscript. PP: acquisition of data; drafting the manuscript. JG: analysis of data; revising the manuscript. All authors read and approved the final manuscript.

## References

[B1] Cobb AR Murthy R Cousin GC El-Rasheed A Toma A Uddin J Manisali M Silent sinus syndrome Br J Oral Maxillofac Surg 2011 11 e81 e85 22051178 10.1016/j.bjoms.2011.10.001

[B2] Montgomery WW Mucocele of the maxillary sinus causing enophthalmos Eye Ear Nose Throat Mon 1964 11 41 44 14143777

[B3] Soparkar CN Patrinely JR Cuaycong MJ Dailey RA Kersten RC Rubin PA Linberg JV Howard GR Donovan DT Matoba AY The silent sinus syndrome. A cause of spontaneous enophthalmos Ophthalmology 1994 11 772 778 8152774 10.1016/s0161-6420(94)31267-x

[B4] Illner A Davidson HC Harnsberger HR Hoffman J The silent sinus syndrome: clinical and radiographic findings AJR Am J Roentgenol 2002 11 503 506 11804926 10.2214/ajr.178.2.1780503

[B5] Habibi A Sedaghat MR Habibi M Mellati E Silent sinus syndrome: report of a case Oral Surg Oral Med Oral Pathol Oral Radiol Endod 2008 11 e32 e35 18280943 10.1016/j.tripleo.2007.09.018

[B6] Rose G Sandy C Hallberg L Moseley I Clinical and radiologic characteristics of the imploding antrum or “silent sinus” syndrome Ophthalmology 2003 11 811 818 12689908 10.1016/S0161-6420(02)01993-0

[B7] Buono LM The silent sinus syndrome: maxillary sinus atelectasis with enophthalmos and hypoglobus Curr Opin Ophthalmol 2004 11 486 489 15523193 10.1097/01.icu.0000142510.68451.32

[B8] Monos T Levy J Lifshitz T Puterman M The silent sinus syndrome Isr Med Assoc J 2005 11 333 335 15909469

[B9] Annino D Goguen L Silent sinus syndrome Curr Opin Otolaryngol Head Neck Surg 2008 11 22 25 18197017 10.1097/MOO.0b013e3282f2c9aa

[B10] Baghat M Bahgat Y Bahgat A Silent sinus syndrome BMJ Case Rep 2012 10.1136/bcr-2012-007198 10.1136/bcr-2012-007198 PMC4544204 23125298

[B11] Brandt MG Wright ED The silent sinus syndrome is a form of maxillary atelectasis: a systematic review of all reported cases Am J Rhinol 2008 11 68 18284862 10.2500/ajr.2008.22.3118

[B12] Sánchez-Dalmau BF Pascual L Lao X Maiz J Sinus syndrome, an uncommon cause of enophthalmos Arch Soc Esp Oftalmol 2008 11 125 128 18260025 10.4321/s0365-66912008000200012

[B13] Gaudino S Lella GM Piludu F Martucci M Schiarelli C Africa E Salvolini L Colosimo C CT and MRI diagnosis of silent sinus syndrome Radiol Med 2012 11 265 275 22580803 10.1007/s11547-012-0822-x

[B14] Sciaretta V Pasquini E Tesei F Modugno GC Farneti G Endoscopic sinus surgery for the treatment of maxillary sinus atelectasis and silent sinus syndrome J Otolaryngol 2006 11 60 64 10.2310/7070.2005.4098 16527020

[B15] Nkenke E Alexiou C Iro H Amann K Kirchner T Häusler G Neukam FW Holbach LM Management of spontaneous enophthalmos due to silent sinus syndrome: a case report Int J Oral Maxillofac Surg 2005 11 809 811 16157252 10.1016/j.ijom.2005.01.011

[B16] Numa WA Desai U Gold DR Heher KL Annino DJ Silent sinus syndrome: a case presentation and comprehensive review of all 84 reported cases Ann Otol Rhinol Laryngol 2005 11 688 694 16240931 10.1177/000348940511400906

[B17] Ferri A Sesenna E Ferri Teore Bilateral silent sinus syndrome: case report and surgical solution J Oral Maxillofac Surg 2012 11 103 106 10.1016/j.joms.2011.08.008 22030251

[B18] Borges P Branco C Subtil J Endonasal endoscopic repair of the orbital floor defect in the silent sinus syndrome Otolaryngol Head Neck Surg 2006 11 800 802 10.1016/j.otohns.2005.02.008 17071316

[B19] Cardesin A Eseamilla Y Romera M Molina JA Single surgical step for endoscopic surgery and orbital reconstruction of a silent sinus syndrome Acta Otorrinolaringol Esp 2013 11 297 299 22421390 10.1016/j.otorri.2011.12.004

[B20] Chow AW Benninger MS Brook I Brozek JL Goldstein EJ Hicks LA Pankey GA Seleznick M Volturo G Wald ER File TM Jr Infectious diseases society of America: IDSA clinical practice guideline for acute bacterial rhinosinusitis in children and adults Clin Infect Dis 2012 11 e72 e112 22438350 10.1093/cid/cir1043

[B21] Hamilos DL Chronic rhinosinusitis: epidemiology and medical management J Allergy Clin Immunol 2011 11 693 707 21890184 10.1016/j.jaci.2011.08.004

[B22] Bolger WE Woodruff WW Morehead J Parsons DS Maxillary sinus hypoplasia: classification and description of associated uncinate process hypoplasia Otolaryngol Head Neck Surg 1990 11 759 2126098 10.1177/019459989010300516

[B23] Erden T Aktas D Erden G Miman MC Ozturan O Maxillary sinus hypoplasia Rhinology 2002 11 150 153 12357716

[B24] Kass ES Salman S Weber AL Rubin PA Montgomery WW Chronic maxillary atelectasis Ann Oto Rhino Laryngol 1997 11 109 116 10.1177/000348949710600204 9041814

[B25] Price D Friedman O Facial assymetry in maxillary sinus hipoplasia Int J Pediatr Otorhinolaryngol 2007 11 1627 1630 17692391 10.1016/j.ijporl.2007.06.014

[B26] Arikan OK Onaran Z Muluk NB Yilmazbaş P Yazici I Enophthalmos due to atelectasis of the maxillary sinus: silent sinus syndrome J Craniofac Surg 2009 11 2156 2159 19884840 10.1097/SCS.0b013e3181bf0116

[B27] Vander Meer JB Harris G Toohill RJ Smith TL The silent sinus syndrome: a case series and literature review Laryngoscope 2001 11 975 978 11404606 10.1097/00005537-200106000-00008

[B28] Naik RM Khemani S Saleh HA Frontal silent sinus syndrome Otolaryngol Head Neck Surg 2013 11 354 355 23124922 10.1177/0194599812466646

[B29] Jung D Gray S Silent sinus syndrome after lateral fracture of the inferior turbinate Otolaryngol Head Neck Surg 2012 11 863 864 21952356 10.1177/0194599811424041

[B30] Bossolesi P Autelitano L Brusati R Castelnuovo P The silent sinus syndrome: diagnosis and surgical treatment Rhinology 2008 11 308 316 19146002

